# Co-expression effect of LLGL2 and SLC7A5 to predict prognosis in ERα-positive breast cancer

**DOI:** 10.1038/s41598-022-20225-4

**Published:** 2022-10-03

**Authors:** Tomoka Hisada, Naoto Kondo, Yumi Wanifuchi-Endo, Satoshi Osaga, Takashi Fujita, Tomoko Asano, Yasuaki Uemoto, Sayaka Nishikawa, Yusuke Katagiri, Mitsuo Terada, Akiko Kato, Hiroshi Sugiura, Katsuhiro Okuda, Hiroyuki Kato, Masayuki Komura, Satoshi Morita, Satoru Takahashi, Tatsuya Toyama

**Affiliations:** 1 Department of Breast Surgery, Nagoya City University Graduate School of Medical Sciences, 1 Kawasumi, Mizuho-cho, Mizuho-ku, Nagoya, Aichi 467-8601 Japan; 2 Clinical Research Management Center, Nagoya City University Hospital, Nagoya, Aichi Japan; 3 Department of Breast and Endocrine Surgery, Nagoya City University West Medical Center, Nagoya, Aichi Japan; 4 Department of Oncology, Immunology and Surgery, Nagoya City University Graduate School of Medical Sciences, Nagoya, Aichi Japan; 5 Department of Experimental Pathology and Tumor Biology, Nagoya City University Graduate School of Medical Sciences, Nagoya, Aichi Japan; 6 Department of Biomedical Statistics and Bioinformatics, Kyoto University Graduate School of Medicine, Kyoto, Japan

**Keywords:** Breast cancer, Tumour biomarkers

## Abstract

Lethal giant larvae homolog 2 (LLGL2) and solute carrier family 7 member 5 (SLC7A5) have been reported to be involved in resistance to endocrine therapy. This study aimed to assess the effects of LLGL2/SLC7A5 co-expression in predicting prognosis and response to tamoxifen therapy in ERα-positive breast cancer patients according to *LLGL2/SLC7A5* mRNA and protein expression in long-term follow-up invasive breast cancer tissues. We identified that low *LLGL2/SLC7A5* mRNA co-expression (*LLGL2*^low^/*SLC7A5*^low^) was associated with disease-free survival (DFS) compared with other combination groups in all breast cancer patients. In ERα-positive breast cancer patients, *LLGL2*^low^/*SLC7A5*^low^ showed longer DFS and overall survival (OS) compared with *LLGL2*^high^/*SLC7A5*^high^ and a positive trend of longer survival compared with the other combination groups. We also observed that *LLGL2*^low^/*SLC7A5*^low^ showed longer survival compared with *LLGL2*^high^/*SLC7A5*^high^ in ERα-positive breast cancer patients receiving adjuvant tamoxifen therapy. Multivariate analysis demonstrated that *LLGL2*^low^/*SLC7A5*^low^ was an independent favorable prognostic factor of both DFS and OS, not only in all breast cancer patients, but also in ERα-positive breast cancer patients. High co-expression of LLGL2 and SLC7A5 protein showed a positive trend of shorter survival. Our study showed that co-expression of *LLGL2* and *SLC7A5* mRNA is a promising candidate biomarker in early breast cancer patients.

## Introduction

Breast cancer remains the leading cause of cancer-related deaths in women. Approximately 70% of all breast cancer cases express estrogen receptor α (ERα)^1,2^. In ERα-positive breast cancers, estradiol is a critical regulator of cell proliferation and survival. Estradiol directly regulates genes through binding to ERα or indirectly through activating plasma membrane-associated ERα. Treatment options for ERα-positive breast cancer patients include endocrine therapies that inhibit ERα signaling, either by antagonizing ligand binding to ERα, downregulating ERα, or suppressing estrogen production^3^.

Tamoxifen is one of the most common endocrine treatments for breast cancer. Tamoxifen treatment was demonstrated to reduce the risk of breast cancer recurrence and death in ERα-positive breast cancer patients^4^. Although endocrine therapy has dramatically improved survival in ERα-positive breast cancer patients, some tumors show de novo or acquired drug resistance to endocrine therapy^5–7^. Resistance to endocrine therapies including tamoxifen remains a major challenge in the treatment of ERα-positive breast cancer patients. Furthermore, there are few established biomarkers for tamoxifen treatment resistance that have been applied in current clinical practice.

Recently, Saito et al. reported that lethal giant larvae homolog 2 (LLGL2) functions as a promoter of tumor growth in ERα-positive breast cancer^8^. They observed that the intracellular concentration of leucine was decreased in MCF-7 ERα-positive breast cancer cells when *LLGL2* expression was knocked down. They also demonstrated that the proliferation of MCF-7 cells was suppressed when *LLGL2* expression was knocked down, and that excess leucine could rescue the proliferation of *LLGL2*-knockdown cells^8^.

LLGL2 is reported to be localized at cell junctions and membranes with a member of the solute carrier (SLC) family, SLC7A5, which is the primary leucine transporter in cells^8,9^. SLC7A5 is a sodium-independent amino acid transporter that imports leucine^10^. High SLC7A5 expression was reported to be associated with poor prognosis in various cancers including breast cancer^11–15^.

LLGL2 and SLC7A5 are involved in resistance to tamoxifen treatment^8,12,16^. LLGL2 was also reported to function with SLC7A5 at cell junctions and membranes in ERα-positive breast cancer cells, and LLGL2 interacts with SLC7A5 to promote cell proliferation^8^. Therefore, we hypothesized that both LLGL2 and SLC7A5 are required for the efficacy of tamoxifen treatment in ERα-positive breast cancer patients.

In this study, we assessed the effects of LLGL2/SLC7A5 co-expression in predicting prognosis and response to tamoxifen therapy in ERα-positive breast cancer patients with long-term follow up.

## Results

### *LLGL2* mRNA expression and prognosis of breast cancer patients

We first investigated the association between *LLGL2* mRNA expression level and prognosis of breast cancer patients with long-term follow-up. A total of 624 breast cancer tissue samples were subjected to *LLGL2* mRNA expression analysis. The associations between *LLGL2* mRNA expression and clinicopathological characteristics are shown in Supplementary Table S1. Low *LLGL2* mRNA levels were positively associated with larger tumor size (*P* = 0.047) and lymph node negativity (*P* = 0.031). Low *LLGL2* mRNA expression was positively associated with longer DFS in all breast cancer patients analyzed in this study (*P* = 0.023; Supplementary Fig. S1a). Furthermore, patients with tumors showing low *LLGL2* mRNA expression showed a tendency towards longer OS (*P* = 0.072; Supplementary Fig. S1b).

LLGL2 was reported to be involved in prognosis only in ERα-positive breast cancer patients^8^. Therefore, we next investigated the association of *LLGL2* mRNA expression with prognosis according to ERα status. In ERα-positive breast cancer patients, there was a positive correlation between low *LLGL2* mRNA expression and longer DFS and OS (*P* = 0.0009 and *P* = 0.005, respectively; Supplementary Fig. S1c,d); however, in ERα-negative breast cancer patients, there was no association between *LLGL2* mRNA expression and prognosis (Supplementary Fig. S1e,f). The clinicopathological characteristics of ERα-positive breast cancer patients are shown in Supplementary Table S2. Low *LLGL2* mRNA expression level was positively associated with lymph node negativity (*P* = 0.007).

LLGL2 was reported to be involved in resistance to tamoxifen in ERα-positive breast cancer patients^8^. Therefore, we investigated the association of *LLGL2* mRNA expression with prognosis in ERα-positive breast cancer patients receiving adjuvant tamoxifen therapy (n = 272). As shown in Supplementary Fig. S1g,h, positive associations were found between low *LLGL2* mRNA expression and longer DFS and OS in ERα-positive breast cancer patients receiving adjuvant tamoxifen therapy (*P* = 0.016 and *P* = 0.018, respectively). Interestingly, no associations were identified between *LLGL2* mRNA expression level and prognosis in ERα-positive breast cancer patients without adjuvant tamoxifen therapy (Supplementary Fig. S1i,j).

We next performed univariate and multivariate Cox regression analyses of clinicopathological factors associated with prognosis using stepwise linear regression in all breast cancer patients (Supplementary Table S3) and in ERα-positive breast cancer patients analyzed in this study (Supplementary Table S4). Although low *LLGL2* mRNA expression was not an independent favorable prognostic factor in all breast cancer patients, we showed that low *LLGL2* was an independent favorable prognostic factor for both DFS and OS in ERα-positive breast cancer patients, as well as nodal status (*P* = 0.012 and *P* = 0.011, respectively).

### *SLC7A5* mRNA expression and prognosis of breast cancer patients

Next, we investigated the association between *SLC7A5* mRNA expression and the prognosis of breast cancer patients. The characteristics of ERα-positive breast cancer patients according to *SLC7A5* mRNA expression are shown in Supplementary Table S5. Low *SLC7A5* mRNA expression was positively associated with favorable prognosis in both DFS and OS in all breast cancer patients analyzed (*P* = 0.002 and *P* = 0.0005, respectively; Supplementary Fig. S2a,b). Low *SLC7A5* mRNA expression was also positively associated with favorable prognosis in both DFS and OS in ERα-positive breast cancer patients (*P* = 0.004 and *P* = 0.004, respectively; Supplementary Fig. S2c,d), but no association was observed in ERα-negative breast cancer patients (Supplementary Fig. S2e,f). As shown in Supplementary Fig. S2g, positive associations were identified between low *SLC7A5* mRNA expression and longer DFS in ERα-positive breast cancer patients receiving adjuvant tamoxifen therapy (*P* = 0.014).

### Combination of *LLGL2* and *SLC7A5* mRNA expression and prognosis of breast cancer patients

**Figure 1:**
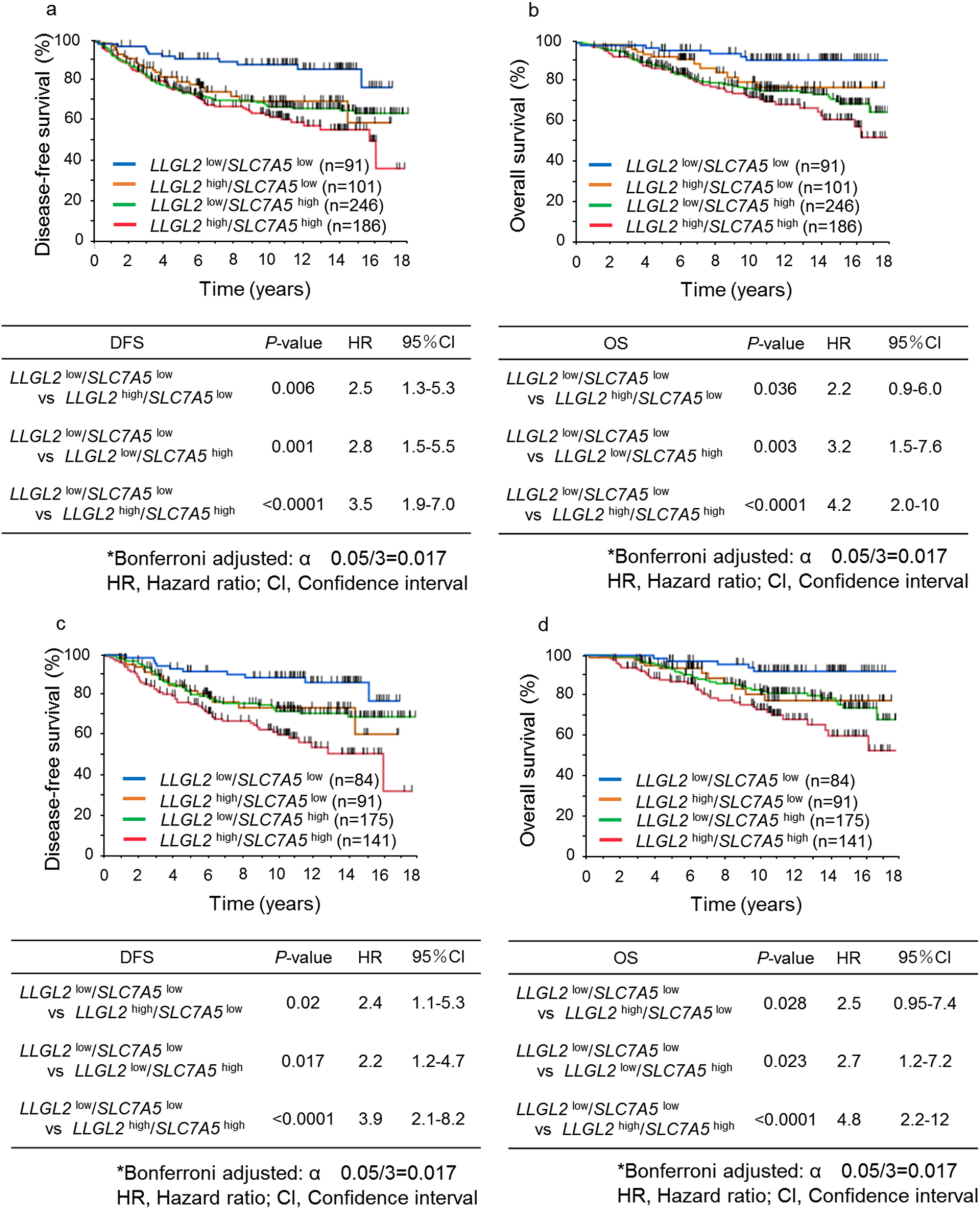

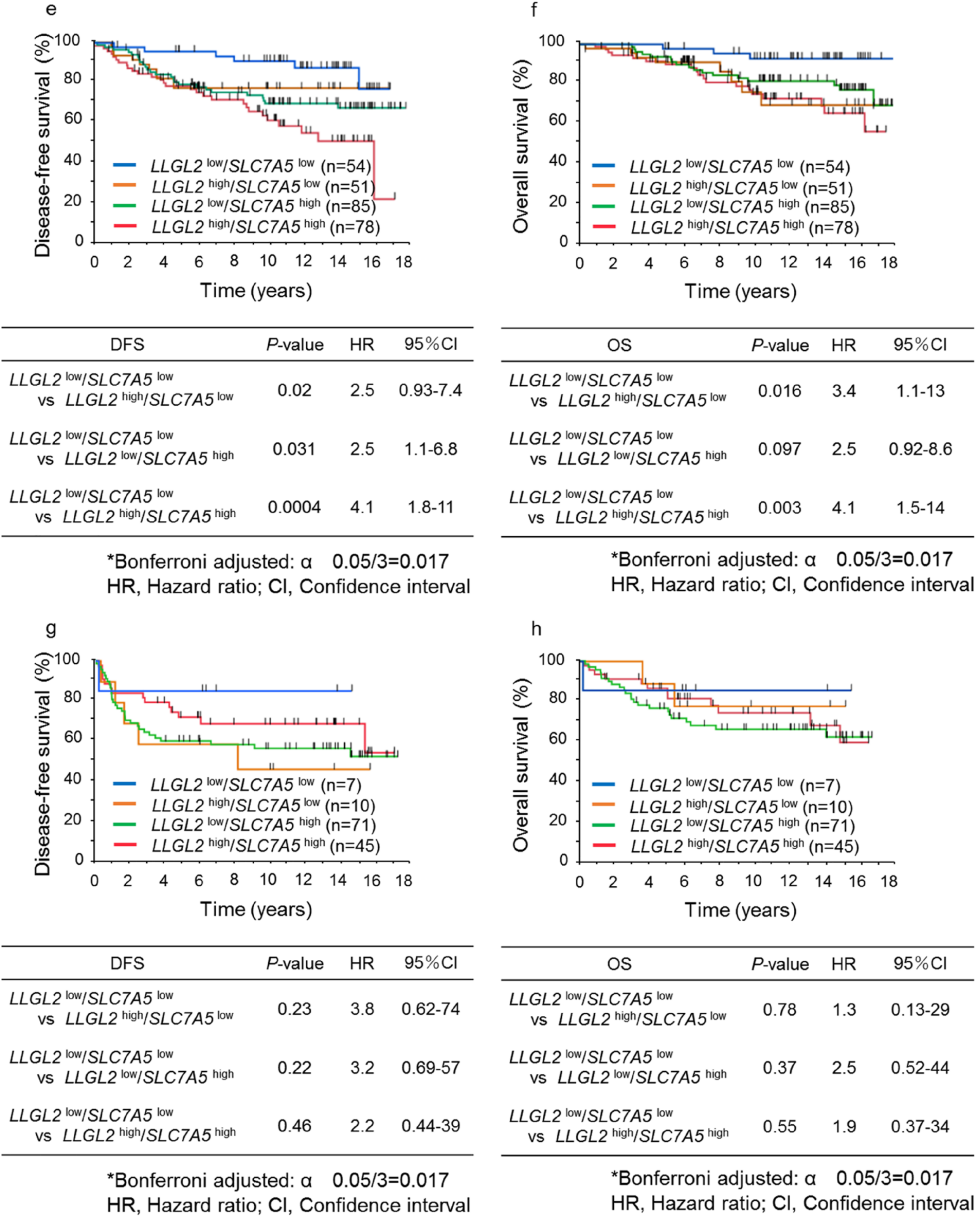
Kaplan–Meier survival curves according to the combination of *LLGL2* and *SLC7A5* mRNA expression. Graphs show DFS and OS curves for all breast cancer patients (**a,b**), ERα-positive breast cancer patients (**c,d**), ERα-positive breast cancer patients receiving adjuvant tamoxifen therapy (**e,f**), and ERα-negative breast cancer patients (**g,h**).

We then investigated the prognostic impact of the combination of *LLGL2* and *SLC7A5* mRNA expression. Supplementary Table S6 shows the characteristics of breast cancer patients classified by the combination of *LLGL*2 and *SLC7A5* mRNA expression. Low *LLGL2*/*SLC7A5* mRNA co-expression (*LLGL2*^low^/*SLC7A5*^low^) was positively associated with lower histological grade, lymph node negativity, and ERα positivity. As shown in Fig. 1a,b, *LLGL2*^low^/*SLC7A5*^low^ was associated with longer survival compared with the other combination groups in all breast cancer patients analyzed in this study. The characteristics of ERα-positive breast cancer patients according to *LLGL2*/*SLC7A5* mRNA co-expression are shown in Table 1. *LLGL2*^low^/*SLC7A5*^low^ was associated with lower grade and lymph node negativity in ERα-positive breast cancer patients. As shown in Fig. 1c,d, *LLGL2*^low^/*SLC7A5*^low^ showed longer survival compared with high *LLGL2*/*SLC7A5* mRNA co-expression (*LLGL2*^high^/*SLC7A5*^high^) and a positive trend of longer survival compared with other combination groups in ERα-positive breast cancer patients. However, we did not observe the association between *LLGL2/SLC7A5* mRNA expression and prognosis in ERα-negative breast cancer patients (Fig. 1g,h). Then, we investigated the association of prognosis with the combination of *LLGL2* and *SLC7A5* mRNA expression in ERα-positive breast cancer patients receiving adjuvant tamoxifen therapy. As shown in Fig. 1e,f, *LLGL2*^low^/*SLC7A5*^low^ showed longer survival than *LLGL2*^high^/*SLC7A5*^high^ and a positive trend of longer survival compared with other combination groups in ERα-positive breast cancer patients receiving adjuvant tamoxifen therapy. No significant difference was observed between these four combination groups in ERα-positive breast cancer patients who had not received adjuvant tamoxifen therapy (Supplementary Fig. S3a,b).

**Table 1:** Association between *LLGL2 and SLC7A5* mRNA expression and clinicopathological characteristics in ERα-positive patients.

	*LLGL2/SLC7A5*				*P* value
	Low/low	High/low	Low/high	High/high	
	n (%)	n (%)	n (%)	n (%)	
Patients	84	91	175	141	
**Menopausal status**					
Pre	44 (52)	43 (47)	75 (43)	54 (38)	0.19
Post	40 (48)	48 (53)	100 (57)	87 (62)	
**Tumor size**					
≤ 2 cm	29 (35)	45 (50)	71 (40)	62 (44)	0.22
> 2 cm	55 (65)	46 (50)	104 (60)	79 (56)	
Unknown	0	0	0	0	
**Nodal status**					
Negative	56 (66)	50 (55)	95 (54)	57 (40)	0.001
Positive	24 (29)	37 (41)	72 (41)	76 (54)	
Unknown	4 (5)	4 (4)	8 (5)	8 (6)	
**Grade**					
1 + 2	64 (76)	64 (70)	101 (58)	86 (61)	0.011
3	18 (22)	25 (28)	71 (40)	50 (35)	
Unknown	2 (2)	2 (2)	3 (2)	5 (4)	
pStage TNM*					
I	27 (32)	30 (33)	51 (29)	36 (26)	0.80
II	44 (52)	43 (47)	83 (47)	71 (50)	
III	9 (11)	13 (14)	33 (19)	24 (17)	
Unknown	4 (5)	5 (5)	8 (5)	10 (7)	
**Histology**					
IDC	73 (88)	79 (85)	160 (91)	126 (89)	0.35
ILC	4 (4)	6 (7)	5 (3)	11 (8)	
Others	6 (7)	6 (7)	10 (6)	4 (3)	
Unknown	1 (1)	0	0	0	
**PgR status**					
Positive	76 (85)	76 (90)	155 (89)	119 (84)	0.39
Negative	8 (15)	15 (10)	20 (11)	22 (16)	
Unknown	0	0	0	0	
**HER2 status**					
Positive	6 (7)	11 (12)	15 (9)	13 (9)	0.73
Negative	75 (89)	78 (86)	152 (87)	124 (88)	
Unknown	3 (4)	2 (2)	8 (4)	4 (3)	
**Adjuvant therapy**					
ET alone	56 (67)	47 (52)	85 (49)	63 (45)	
CT alone	3 (4)	1 (1)	19 (10)	6 (4)	
ET + CT	23 (27)	34 (37)	54 (31)	62 (44)	
None	2 (2)	8 (9)	12 (7)	9 (6)	
Unknown	0	1 (1)	5 (3)	1 (1)	

*PgR* progesterone receptor, *IDC* invasive ductal carcinoma, *ILC* invasive lobular carcinoma, *HER2* human epidermal growth factor receptor 2, *ET* endocrine therapy, *CT* chemotherapy.

*AJCC stage.

**Figure 2:**
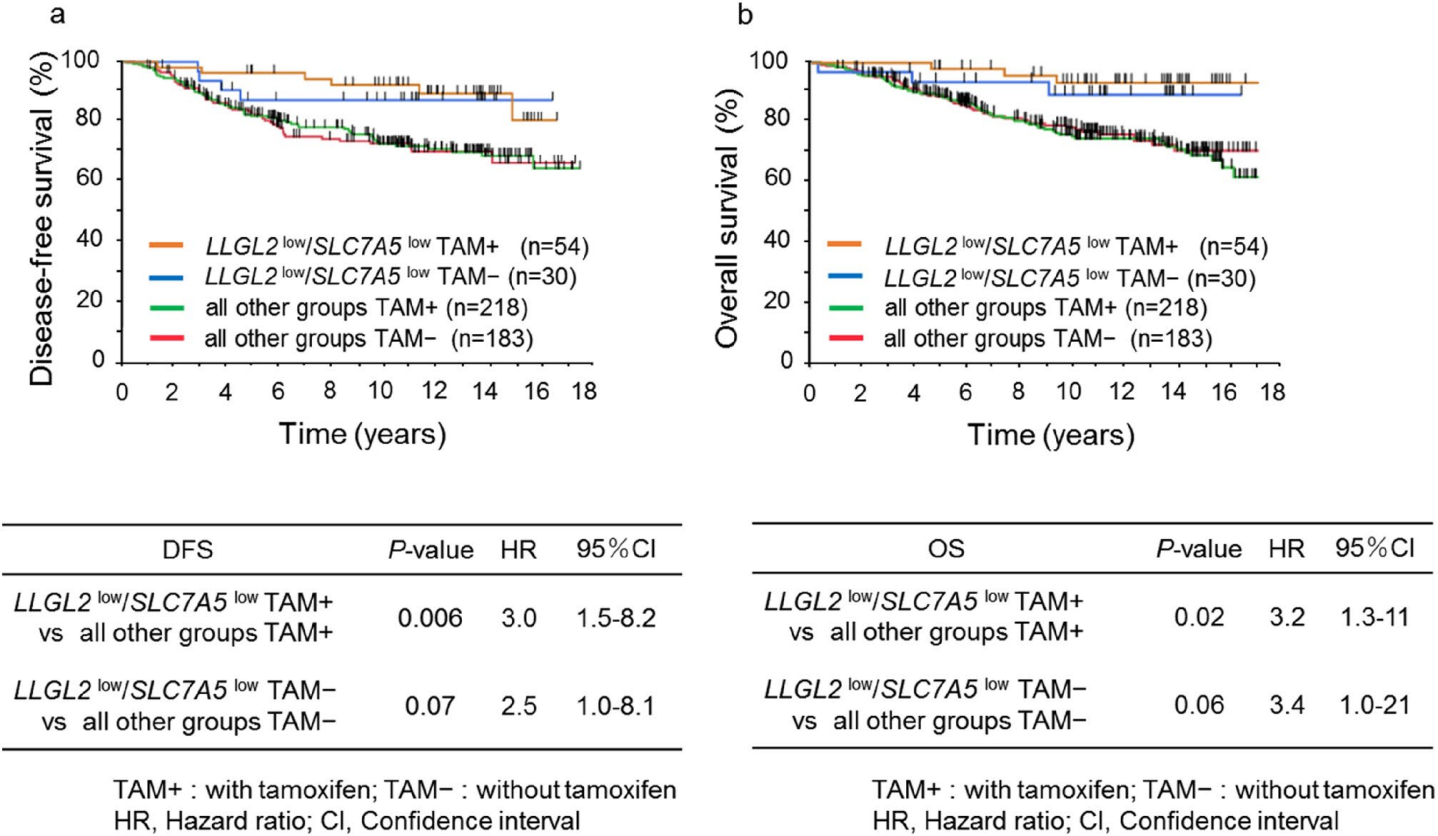
Kaplan–Meier survival curves according to *LLGL2*^low^/*SLC7A5*^low^ versus all other groups with and without adjuvant tamoxifen therapy. Graphs show DFS and OS curves (**a,b**).

Next, we investigated the prognostic value of *LLGL2*^low^/*SLC7A5*^*l*ow^ versus all other groups including *LLGL2*^high^/*SLC7A5*^low^, *LLGL2*^low^/*SLC7A5*^high^, and *LLGL2*^high^/*SLC7A5*^high^ in patients who had received tamoxifen as an adjuvant therapy versus those who had not. As shown in Fig. 2, patients with tumors showing *LLGL2*^low^/*SLC7A5*^low^ who had received tamoxifen as an adjuvant therapy (*LLGL2*^low^ /*SLC7A5*^low^ TAM+) showed not only significantly better DFS, but also significantly better OS compared with the patients of all other groups who had received tamoxifen as an adjuvant therapy (all other groups TAM+) (*P* = 0.006 and *P* = 0.02, respectively). However, there was no significant difference in DFS and OS between the patients with tumors showing *LLGL2*^low^/*SLC7A5*^low^ who had not received tamoxifen as an adjuvant therapy (*LLGL2*^low^/*SLC7A5*^low^ TAM−) compared with the patients of all other groups who had not received tamoxifen as an adjuvant therapy (all other groups TAM−).

We performed univariate and multivariate Cox regression analyses of clinicopathological factors associated with prognosis using stepwise linear regression in each group of breast cancer patients. Multivariate analyses demonstrated that *LLGL2*^low^/*SLC7A5*^low^ was an independent favorable prognostic factor for DFS as well as lymph node negativity and ERα positivity in all analyzed breast cancer patients (Supplementary Table S7). Then, we performed univariate and multivariate analyses in ERα-positive breast cancer patients, which identified *LLGL2*^low^/*SLC7A5*^low^ as an independent favorable prognostic factor for both DFS and OS, as well as lymph node negativity (Table 2).

**Table 2:** Univariate and multivariate analyses of factors associated with DFS and OS including *LLGL2/SLC7A5* in ERα-positive patients.

Variate	n (%)	Univariate (DFS) (DFS) (DFS)	Univariate (OS) (OS)	Multivariate (DFS) (DFS)		Multivariate (OS) (OS)	
		*P*-value	*P*-value	*P*-value	HR (95% CI)	*P*-value	HR (95% CI)
**Menopausal status**							
Pre	206 (47)						
Post	237 (53)	0.29	0.20				
**Tumor size**							
≤ 2 cm	184 (42)						
> 2 cm	259 (58)	0.14	0.011				1 (Reference)
**Nodal status**						0.045	1.72 (1.01, 2.94)
Negative	244 (55)				1 (Reference)		1 (Reference)
Positive	199 (45)	< 0.0001	< 0.0001	< 0.0001	2.90 (1.93, 4.35)	0.0001	2.68 (1.62, 4.44)
**Grade**							
1 and 2	287 (65)						
3	156 (35)	0.39	0.11				
**Histology**							
IDC	402 (91)						
ILC	18 (4)	0.59	0.37				
Others	23 (5)	0.095	0.29				
**HER2 status**							
Negative	402 (91)				1 (Reference)		1 (Reference)
Positive	41 (9)	0.19	0.038	0.054	1.78 (0.99, 3.20)	0.004	2.64 (1.37, 5.10)
***LLGL2/SLC7A5***							
Low/low	76 (17)				1 (Reference)		1 (Reference)
High/low	84 (19)	0.018	0.043	0.04	2.53 (1.04, 6.14)	0.047	3.28 (1.02, 10.58)
Low/high	157 (35)	0.008	0.012	0.022	2.57 (1.15. 5.75)	0.02	3.47 (1.22, 9.90)
High/high	126 (28)	< 0.0001	0.0004	0.0008	3.97 (1.78, 8.85)	0.001	5.58 (1.94, 16.02)

*IDC* invasive ductal carcinoma, *ILC* invasive lobular carcinoma, *HER2* human epidermal growth factor receptor 2.

### Protein expression of LLGL2 and SLC7A5 in breast cancer patients

**Figure 3:**
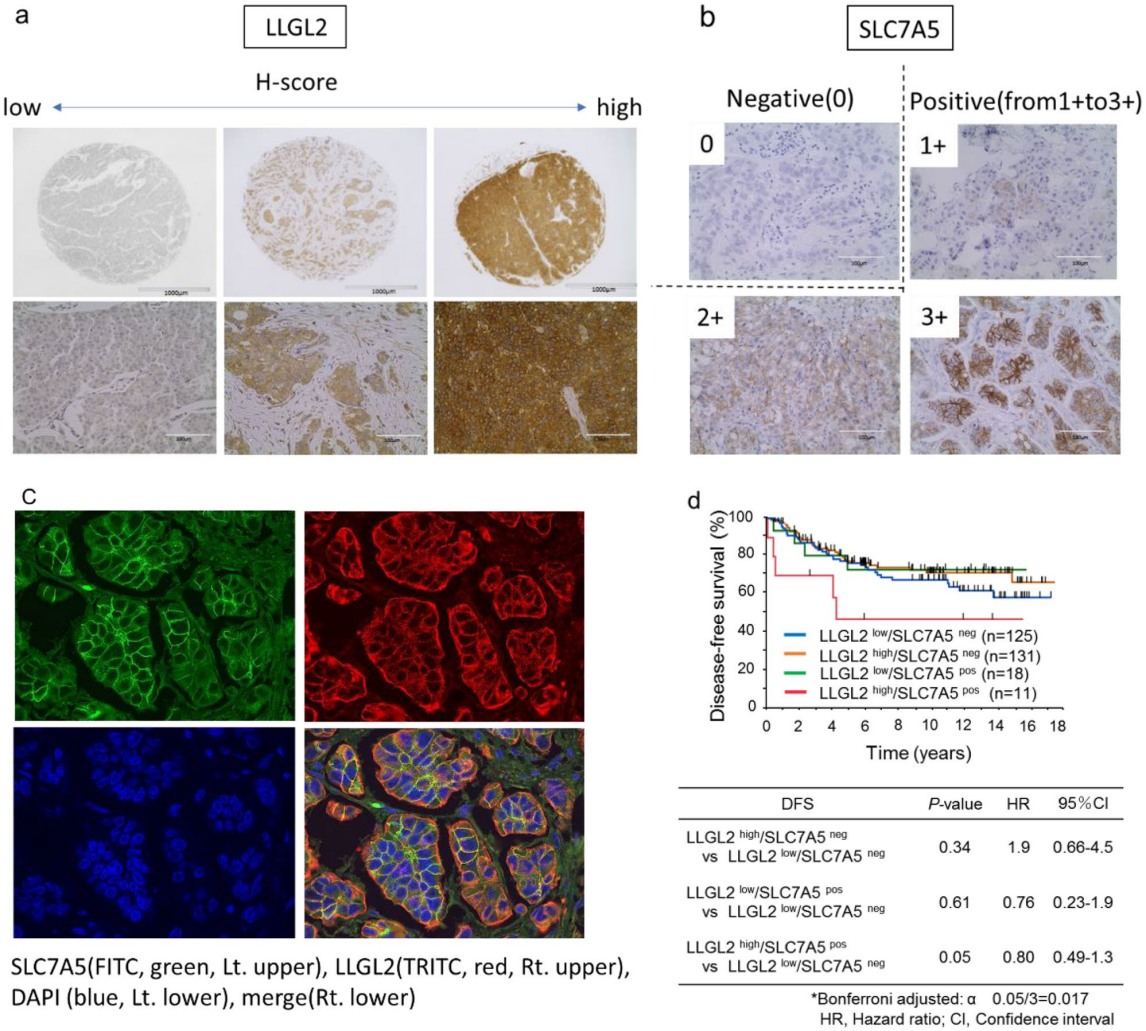
Representative images of LLGL2 protein expression in breast cancer tissues. H-score: 0, 90, and 270 from the left (**a**). Representative images of SLC7A5 protein expression in breast cancer tissues. 0 is negative and 1+ to 3+ is positive (**b**). Immunofluorescence staining for LLGL2 and SLC7A5. SLC7A5 (FITC, green, lt. upper), LLGL2 (TRITC, red, rt. upper), DAPI (blue, lt. lower), and merge (rt. lower) (**c**). Kaplan–Meier survival curves of DFS according to the combination of LLGL2 and SLC7A5 protein expression (**d**).

The expression levels of LLGL2 protein in breast cancer tissue samples were examined by Immnohistochemistry (IHC). LLGL2 protein expression was observed in the cytoplasm. Representative images of LLGL2 and SLC7A5 are shown in Fig. 3a,b. Immunostaining results were evaluated using the Aperio scanscopeCS2 and eSlide manager application, and H-scores were calculated by this digital pathological system. In this study, a total of 285 consecutive breast cancer tissue samples for which mRNA expression data were available were analyzed for LLGL2 protein expression.

We also evaluated the expression levels of SLC7A5 protein in breast cancer tissue samples. The analysis was performed using the same tissue microarray as the protein expression analysis of LLGL2. SLC7A5 protein expression was observed in the cell membrane (Fig. 3a). Furthermore, SLC7A5 protein expression was observed in 10% of breast cancer tissues.

We then performed immunofluorescence to further clarify the localization of each protein. As shown in Fig. 3c, LLGL2 was expressed in the cytoplasm and SLC7A5 in the plasma membrane, indicating co-expression in the same tumor cells. Next, we investigated the association between prognosis and the combination of LLGL2 and SLC7A5 protein expression. The LLGL2^high^/SLC7A5^pos^ group seemed to show the worst prognosis among the four groups, although there was no statistically significant difference between them (Fig. 3d). The median H-score was used as the cutoff value for LLGL2, and SLC7A5 was divided into two groups based on the presence or absence of staining.

## Discussion

In this study, we evaluated the prognostic value of co-expression of LLGL2 and SLC7A5 in primary breast cancer patients with long-term follow-up. First, we showed that low *LLGL2* or low *SLC7A5* mRNA expression was an independent favorable prognostic factor in Erα-positive breast cancer patients. Second, we demonstrated that low *LLGL2*/*SLC7A5* mRNA co-expression (*LLGL2*^low^/*SLC7A5*^low^) was also an independent favorable prognostic factor both in all breast cancer patients and in Erα-positive breast cancer patients. We also observed that *LLGL2*^low^/*SLC7A5*^low^ showed longer survival compared with *LLGL2*^high^/*SLC7A5*^high^ and a positive trend of longer survival compared with other combination groups in Erα-positive breast cancer patients receiving adjuvant tamoxifen therapy.

Saito et al*.* recently reported that LLGL2 promoted leucine uptake and conferred tumor growth and resistance to tamoxifen treatment by increasing the cell surface levels of SLC7A5 in Erα-positive breast cancer. They also reported that low *LLGL2* mRNA expression was a favorable prognostic factor in Erα-positive breast cancer^8^. In this study, our data supported the report by Saito et al.^8^. However, in Erα-negative breast cancer, high expression of *LLGL2* mRNA was a favorable prognostic trend. This result was also consistent with previous reports, suggesting that the function of LLGL2 differs depending on the ER status of the tumor.

In this study, we showed that low *SLC7A5* mRNA expression was positively associated with favorable prognosis in Erα-positive breast cancer patients but not in Erα-negative breast cancer patients. Our data in this study supported the previous report by Ansari et al*.*^11^. Although SLC7A5 is a systemic L-amino acid transporter that carries branch-chain amino acids such as leucine and bulky amino acids such as glutamine, which are considered master regulators of the mTORC1 signaling pathway^17,18^, the mechanism by which SLC7A5 affects the prognosis of Erα-positive breast cancer patients is not yet fully understood. Recently, Ansari et al*.* reported that enhanced glutamine uptake by SLC family members including SLC7A5 affects the composition of immune cell infiltrates and might be involved in breast cancer progression^19,20^.

In this study, we demonstrated that *LLGL2*^low^/*SLC7A5*^low^ was an independent favorable prognostic factor not only in all breast cancer patients, but also in Erα-positive breast cancer patients. In Erα-positive breast cancer patients receiving adjuvant tamoxifen therapy, we observed that *LLGL2*^low^/*SLC7A5*^low^ showed longer survival compared with *LLGL2*^high^/*SLC7A5*^high^ and a positive trend of longer survival compared with other combination groups. However, no significant difference was observed between these four combination groups in Erα-positive breast cancer patients who had not received adjuvant tamoxifen therapy, although it should be noted that the small sample size resulted in decreased power. Moreover, we showed that patients with tumors showing *LLGL2*^low^/*SLC7A5*^low^ who had received tamoxifen as an adjuvant therapy were significantly associated with better prognosis compared with the patients of all other groups who had received tamoxifen as an adjuvant therapy. Saito et al. reported a novel mechanism by which LLGL2 interacts with its cargo, SLC7A5, in the cytoplasm and transports it to the membrane, increasing SLC7A5 levels on the cell surface in Erα-positive breast cancer. They also reported that knockdown of *LLGL2* decreased cell surface levels of SLC7A5, and that knockdown of *LLGL2* or *SLC7A5* was sufficient to restore tamoxifen sensitivity to tamoxifen-resistant Erα-positive breast cancer cells under low leucine concentrations. Our data and that of other groups suggested that co-expression of LLGL2 and SLC7A5 is involved in tamoxifen resistance in Erα-positive breast cancer patients, and that the LLGL2–SLC7A5 axis may be an important determinant of the therapeutic effect of tamoxifen in ER-positive breast cancer.

**Figure 4:**
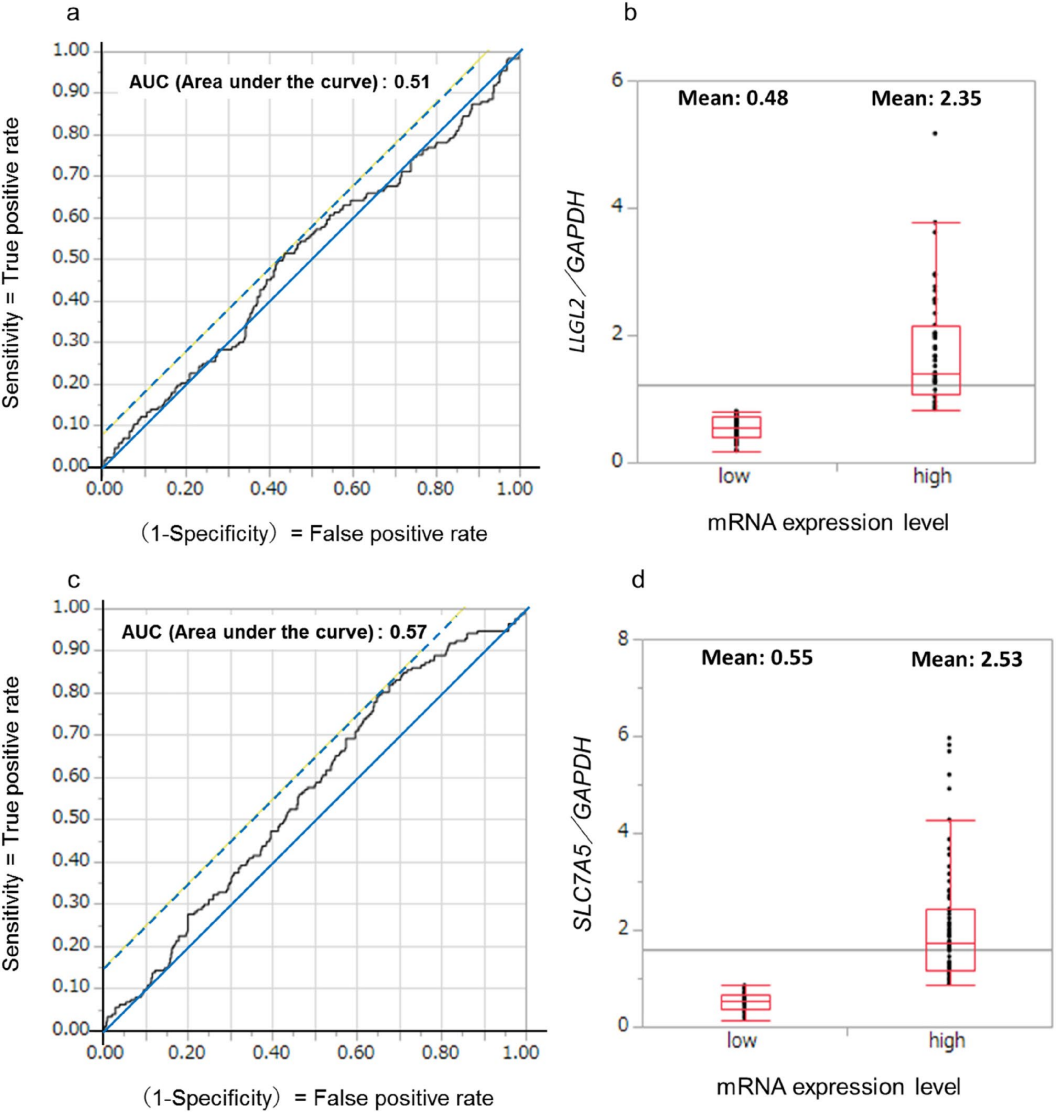
ROC analysis to define the threshold for *LLGL2* and quantitative differences in *LLGL2* mRNA expression (**a,b**). ROC analysis to define the threshold for *SLC7A5* and quantitative differences in *SLC7A5* mRNA expression (**c,d**).

This study had some limitations. First, this was a retrospective analysis at a single institute using archived materials. For the mRNA analysis, we used samples from surgical specimens that were macro-dissected and cryopreserved immediately after resection. However, we did not confirm the amount of cancer tissue in the cryopreserved samples; therefore, the percentage of cancer cells was likely to vary. A total of 626 consecutive invasive breast cancer tissue samples collected between 1992 and 2008 from the archive of our institute were included in this study, and adjuvant therapies for breast cancer have progressed during this period. Therefore, we could not eliminate the effects of different adjuvant therapies in this study. Second, we determined the mRNA cutoff values of *LLGL2* and *SLC7A5* by Receiver Operating Characteristic (ROC) curve analysis (Fig. 4a–d). As a result of these analyses, we determined 0.51 and 0.57 as the cut-off levels of relative *LLGL2* and *SLC7A5* mRNA expression, respectively. Because AUC values of 0.51 and 0.57 do not indicate good discriminatory power, the cut-off value for both mRNAs should be re-evaluated using a different dataset in the future. Third, we did not identify a positive association between LLGL2/SLC7A5 protein expression and prognosis in this study. The positive rate of SLC7A5 was only 10% in this study. Because long-term follow-up tissues were used in this study, the positive rate of staining might have decreased as a result of tissue deterioration over time, but the prognosis of cases with staining was poor, which was consistent with the mRNA results of this study.

In summary, we showed that low *LLGL2/SLC7A5* mRNA co-expression (*LLGL2*^low^/*SLC7A5*^low^) was an independent favorable prognostic factor in ERα-positive breast cancer patients, as well as low *LLGL2* or low *SLC7A5* mRNA expression. We also observed that *LLGL2*^low^/*SLC7A5*^low^ showed longer survival compared with *LLGL2*^high^/*SLC7A5*^high^ and a positive trend of longer survival compared with other combination groups in ERα-positive breast cancer patients receiving adjuvant tamoxifen therapy. Thus, our study showed that the co-expression of *LLGL2* and *SLC7A5* mRNA is a promising candidate biomarker and suggested that the LLGL2–SLC7A5 axis might be a therapeutic target in early breast cancer patients, especially in those receiving adjuvant tamoxifen therapy.

## Methods

### Patients and samples

A total of 626 consecutive invasive breast cancer tissue samples excluding Stage IV collected between 1992 and 2008 from the archive of the Department of Breast Surgery, Nagoya City University Hospital, Japan, were included in this study to measure *LLGL2* and *SLC7A5* mRNA expression. Furthermore, 415 consecutive invasive breast cancer tissues collected between 2000 and 2009 as tissue microarrays were included to evaluate LLGL2 and SLC7A5 protein expression. We performed a statistical analysis of protein expression in 285 of the 415 cases for which mRNA expression data were concurrently available. The tissues were fixed in 10% buffered formalin and embedded in paraffin to make a pathological diagnosis or snap-frozen in liquid nitrogen immediately after resection and stored at − 80 °C until RNA extraction. The histological grade was estimated according to the Bloom and Richardson method proposed by Elston and Ellis^21^. Disease-free survival (DFS) was defined as the interval from the date of primary surgery to the earliest occurrence of one of the following: locoregional recurrence, distant metastasis, or death from any cause. Overall survival (OS) was defined as the interval from the date of primary surgery to death from any cause. The median follow-up period was 10.1 years (range 0.2–215.1 months) and 9.7 years (range 0.8–215.1 months) for the mRNA and protein expression analyses, respectively. Written informed consent for comprehensive research use was obtained from all patients before surgery. This protocol was approved by the institutional review board of Nagoya City University Graduate School of Medical Sciences and conformed to the guidelines of the Declaration of Helsinki.

### RNA extraction and quantitative real-time reverse transcription polymerase chain reaction (RT-PCR)

We used an miRNeasy Mini Kit (QIAGEN, Hilden, Germany) for total RNA extraction from breast cancer tissues and a High-Capacity cDNA Reverse Transcription Kit (Applied Biosystems) for reverse transcription according to each manufacturer’s protocol^22^. mRNA expression levels of *LLGL2*, *SLC7A5*, and the reference gene glyceraldehyde-3-phosphate dehydrogenase (*GAPDH*) were measured using TaqMan Gene Expression assays (Thermo Fisher Scientific). Duplex quantitative RT-PCR assays were performed using the StepOnePlus real-time PCR system (Thermo Fisher Scientific). The reaction was analyzed using a FAM-labeled probe for *LLGL2* or *SLC7A5* (Thermo Fisher Scientific) and a VIC-labeled probe for *GAPDH* (Thermo Fisher Scientific) as a single assay for each sample. The compositions of the amplification reaction mixtures and reaction conditions were as previously reported^22^.

We performed a ROC curve analysis to determine the cut-off values of *LLGL2* and *SLC7A5* mRNA expression (Supplementary Figs. S4, S5). We used Youden’s index (sensitivity + specificity − 1), which corresponded to a point on the ROC curve with the highest vertical distance from the 45° diagonal line, and determined the cut-off values for *LLGL2* and SLC7A5 mRNA expression to be 0.82 and 0.54, respectively.

### IHC of ERα, progesterone receptor (PgR), and human epidermal growth factor receptor 2 (HER2)

One 4-μm-thick section from each paraffin-embedded specimen was first stained with hematoxylin and eosin to ascertain whether an adequate number of invasive ductal carcinoma cells were present and that the quality of fixation was adequate for immunohistochemical analysis. Serial sections (4-μm-thick) were then prepared from suitable tissue blocks and float-mounted on adhesive-coated glass slides for ERα (Dako Envision FLEX-ER, EP1; Agilent Technologies, Santa Clara, CA, USA), PgR (Dako Envision FLEX-ER, PgR636; Agilent Technologies), and HER2 (HercepTest II; Agilent Technologies) staining. Staining of these hormone receptors was performed using the Autostainer Link 48 (Agilent Technologies). Immunostained specimens were scored after the entire section had been evaluated by light microscopy. ERα and PgR expression was evaluated by the percentage of cells with positive nuclear staining. A nuclear staining ratio of ≥ 1/100 was considered positive. Scoring of HER2 expression was based on the membrane staining pattern and was scored on a scale of 0–3 + . Tumors with scores of 0 or 1 were considered negative for HER2 overexpression, and those with a score of 3 were considered positive. Tumors with a score of 2+ were tested for gene amplification by fluorescence in situ hybridization (FISH) using the PathVysion assay (Vysis; Abbott Laboratories, Abbott Park, IL, USA) in accordance with the manufacturer’s protocol. A ratio of > 2.0 for HER2 (*ERBB2*) gene/chromosome 17 was considered positive. Tumors were considered HER2-positive if immunohistochemical staining was 3+ or positive by FISH.

### IHC of LLGL2 and SLC7A5

For the immunohistochemical analysis of LLGL2 and SLC7A5, tissue microarrays on 2-mm-diameter slides were prepared after confirming whether an appropriate number of invasive ductal carcinoma cells was present and whether the fixation quality was suitable for immunohistochemical analysis. The primary antibodies for LLGL2 and SLC7A5 protein were rabbit monoclonal anti-LLGL2 antibody (1:300; Santa Cruz Biotechnology, CA, USA) and rabbit monoclonal anti-SLC7A5/LAT1 antibody (1:100; Abcam, Cambridge, UK). Immunostaining was performed using the Leica Bond-Max automated system and Leica Refine detection kits (Leica Biosystems).

Tissue microarray slides were scanned at × 20 magnification using an Aperio scanscopeCS2 (Leica Biosystems, San Diego, CA, USA). We evaluated at least 1000 tumor cells in each tissue core. The protein expression level of LLGL2 was evaluated according to H-score using the eSlide manager application, a digital pathological system (Leica Biosystems). H-score was calculated by classifying the immunostaining intensity into three categories (weak staining, 1; moderate, 2; and strong, 3) and adding the evaluation of each staining proportion. The threshold optical density for each staining intensity was defined as 210, 180, and 150, respectively. H-score was assigned using the following formula: [1 × (% cells with category 1) + 2 × (% cells with category 2) + 3 × (% cells with category 3)]^23,24^.

SLC7A5 protein expression was localized to the cell membrane, and therefore SLC7A5 immunostaining was evaluated in accordance with the same method employed by the HercepTest (Dako) for HER2 immunostaining, as described above. Scoring of SLC7A5 expression was based on the membrane staining pattern and scored on a scale of 0–3+^25,26^. For immunostaining evaluation, LLGL2 was divided into two groups (high/low) with the median H-score as the cutoff, and SLC7A5 was divided into two groups with 0 as negative and 1+ to 3+ as positive.

### Immunofluorescence staining for LLGL2 and SLC7A5

The detailed methods for immunofluorescence staining employed in this study have been described previously^27^. Frozen sections were cut to 4-μm thickness and fixed in cold acetone and 10% buffered formalin. A mouse monoclonal anti-LLGL2 antibody (Abnova Corporation, Taipei, Taiwan) was used with biotin-conjugated anti-mouse IgG and TRITC-labeled streptavidin (Thermo Fisher Scientific) to visualize the endogenous proteins using an image analyzer (Keyence). A rabbit monoclonal anti-SLC7A5/LAT1 antibody (Abcam, Cambridge, UK) was used with biotin-conjugated anti-rabbit IgG and FITC-labeled streptavidin (Thermo Fisher Scientific).

### Statistical analysis

The associations of *LLGL2* and *SLC7A5* mRNA expression with clinicopathological factors were assessed by χ^2^ and Fisher’s exact probability tests. Survival curves were analyzed using the Kaplan–Meier method and verified by the log-rank test. DFS was censored at the date of last follow-up if patients were still relapse-free and alive, and OS was censored at the time when patients were alive. Cox proportional hazards regression analysis was used for univariate and multivariate analyses of prognostic values using the stepwise variable selection method. The level of statistical significance was set at a *P*-value of less than 5%. Multiple survival curves were compared by the log-rank test with Bonferroni adjustment. In this study, there were three comparisons: *LLGL2*^high^/*SLC7A5*^high^ vs *LLGL2*^low^/*SLC7A5*^low^; *LLGL2*^high^/*SLC7A5*^low^ vs *LLGL2*^low^/*SLC7A5*^low^; and *LLGL2*^low^/*SLC7A5*^high^ vs *LLGL2*^low^/*SLC7A5*^low^. Therefore, a two-sided *P*-value of 0.017 (0.05/3) was taken to indicate statistical significance. Missing data points were excluded from the analysis. Statistical calculations were performed with JMP12.2 software (SAS Institute, Inc., Cary, NC, USA).

### Ethics declarations

This study was approved by the institutional review board of Nagoya City University Graduate School of Medical Sciences. All tissue samples were provided from a biobank that is maintained by the Department of Breast Surgery, Nagoya City University Graduate School of Medical Sciences and conformed to the guidelines of the Declaration of Helsinki. Written informed consent for comprehensive research use was obtained from all patients involved in the study.

## Supplementary Information

Supplementary Figures.

Supplementary Tables.

## Data Availability

The datasets used and/or analyzed during the current study are available from the corresponding author on reasonable request.

## Abbreviations

ERα: Estrogen receptor α
LLGL2: Lethal giant larvae homolog 2
SLC7A5: Solute carrier family 7 member 5
DFS: Disease-free survival
OS: Overall survival
ROC: Receiver operating characteristic
IHC: Immunohistochemistry
PgR: Progesterone receptor
HER2: Human epidermal growth factor receptor 2
OD: Optical density
