# Supplementary material for: Co-expression effect of LLGL2 and SLC7A5 to predict prognosis in ERα-positive breast cancer
Source: Sci Rep. 2022 Oct 3;12:16515. doi: 10.1038/s41598-022-20225-4 (PMC9529905; doi:10.1038/s41598-022-20225-4)
Supplement: Supplementary file 1 — Supplementary Figures. [file 41598_2022_20225_MOESM1_ESM.pdf]

Supplementary Figures

Title: Co-expression effect of LLGL2 and SLC7A5 to predict prognosis in ERα-positive breast cancer

Author's name: Tomoka Hisada, Naoto Kondo, Yumi Wanifuchi-Endo, Satoshi Osaga, Takashi Fujita, Tomoko Asano, Yasuaki Uemoto, Sayaka Nishikawa, Yusuke Katagiri, Mitsuo Terada, Akiko Kato, Hiroshi Sugiura, Katsuhiro Okuda, Hiroyuki Kato, Masayuki Komura, Satoshi Morita, Satoru Takahashi, and Tatsuya Toyama

Supplementary Figure S1

Kaplan–Meier survival curves according to *LLGL2* mRNA expression levels. Graphs show DFS and OS curves for all breast cancer patients (a, b), ERα-positive breast cancer patients (c, d), ERα-negative breast cancer patients (e, f), ERα-positive breast cancer patients receiving adjuvant tamoxifen therapy (g, h), and ERα-positive breast cancer patients without tamoxifen therapy (i, j).

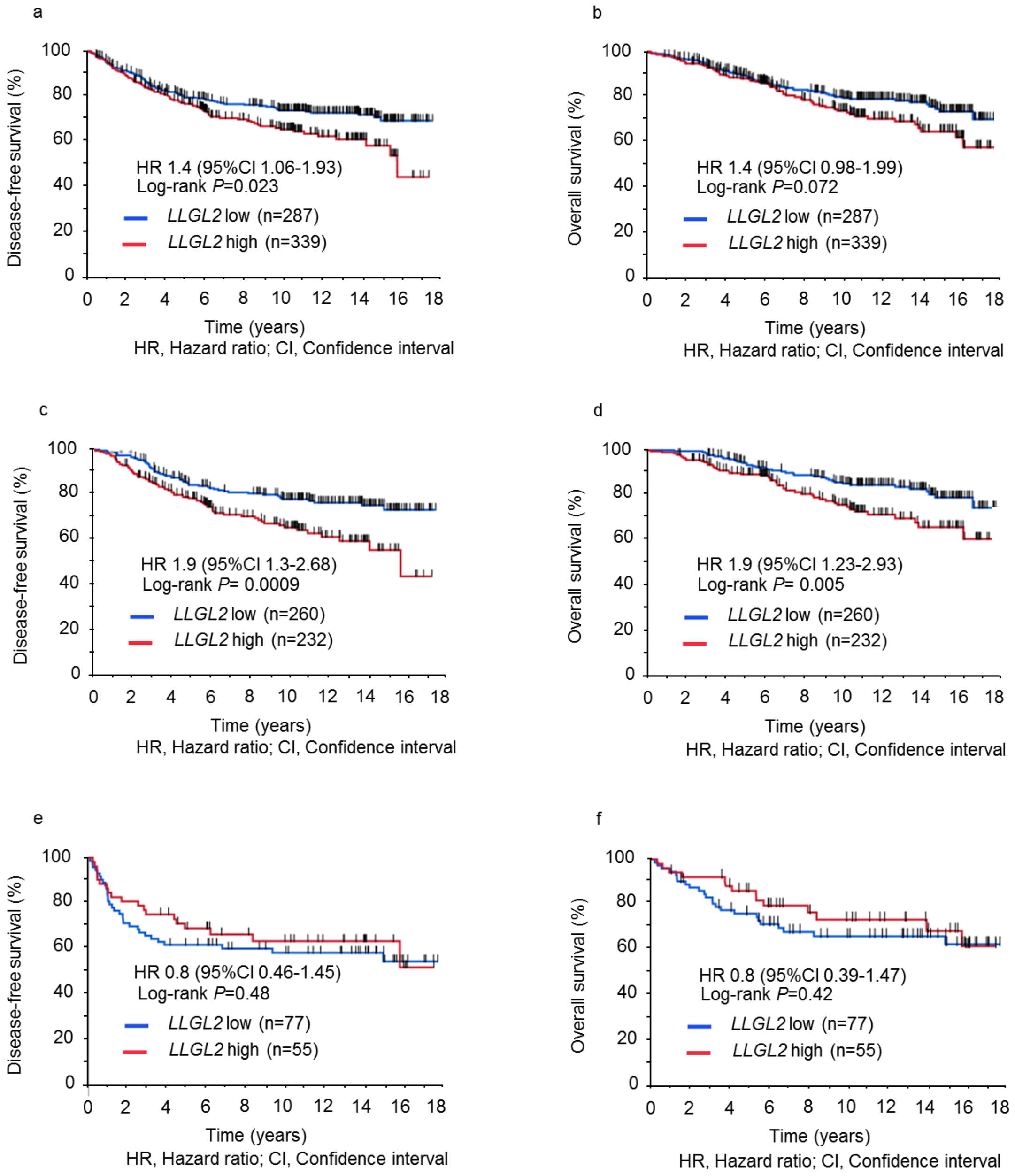

g

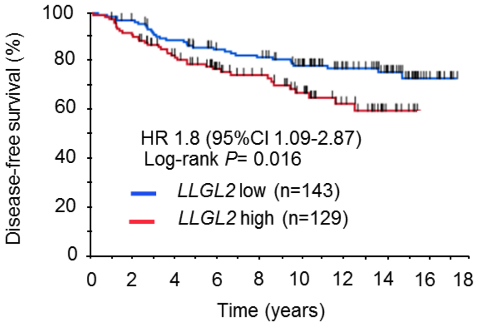

HR, Hazard ratio; CI, Confidence interval

h

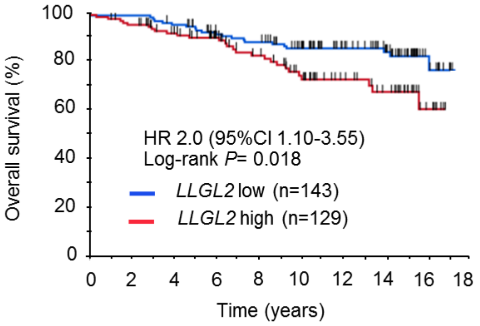

HR, Hazard ratio; CI, Confidence interval

i

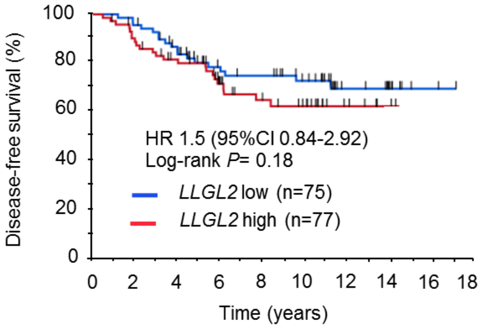

HR, Hazard ratio; CI, Confidence interval

j

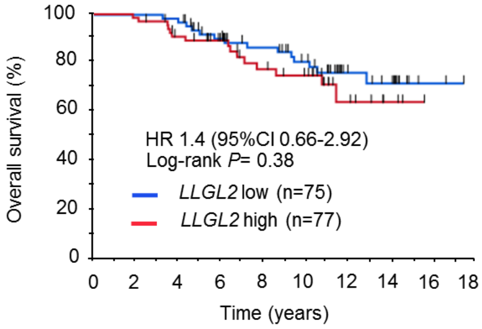

HR, Hazard ratio; CI, Confidence interval

Supplementary Figure S2

Kaplan–Meier survival curves according to *SLC7A5* mRNA expression levels. Graphs show DFS and OS curves for all breast cancer patients (a, b), ERα-positive breast cancer patients (c, d), ERα-negative breast cancer patients (e, f), and ERα-positive breast cancer patients receiving adjuvant tamoxifen therapy (g, h).

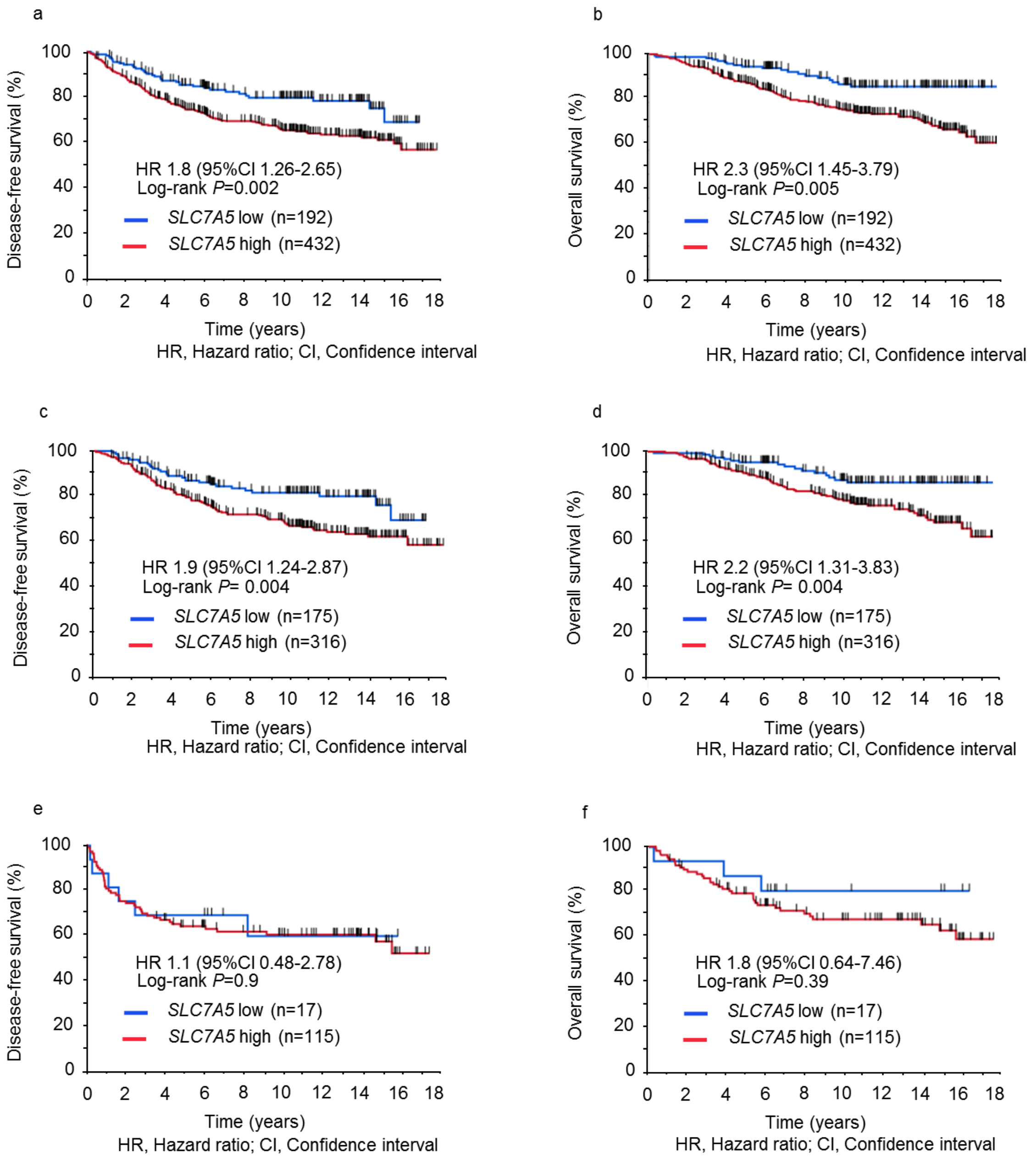

g

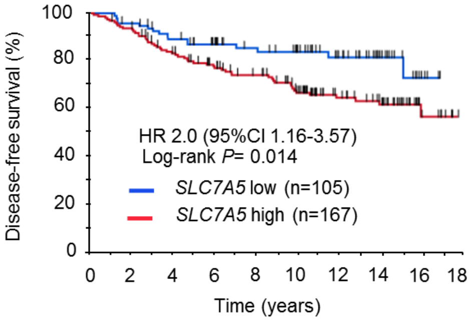

HR, Hazard ratio; CI, Confidence interval

h

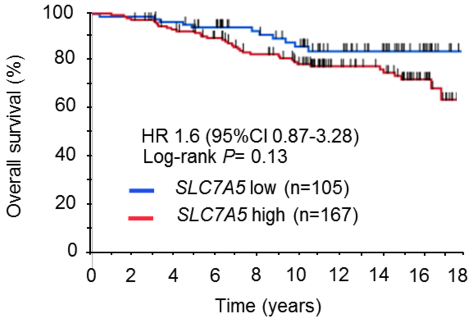

HR, Hazard ratio; CI, Confidence interval

Supplementary Figure S3

Kaplan–Meier survival curves according to the combination of *LLGL2* and *SLC7A5* mRNA expression levels. Graphs show DFS and OS curves for ERα-positive breast cancer patients who had not received adjuvant tamoxifen therapy (a, b).

a

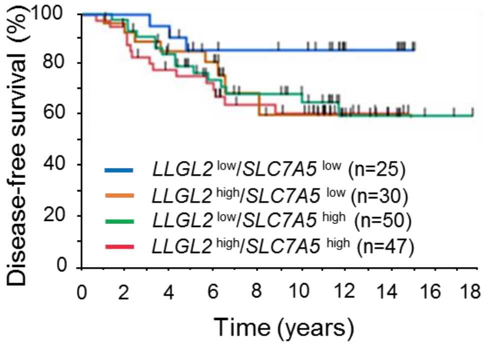

DFS	*P*-value	HR	95%CI
LLGL2 ^{low}/*SLC7A5* ^{low} vs *LLGL2* ^{high}/*SLC7A5* ^{low}	0.068	3.1	0.91-14
LLGL2 ^{low}/*SLC7A5* ^{low} vs *LLGL2* ^{low}/*SLC7A5* ^{high}	0.085	2.8	0.94-12
LLGL2 ^{low}/*SLC7A5* ^{low} vs *LLGL2* ^{high}/*SLC7A5* ^{high}	0.059	3.5	1.1-15

*Bonferroni adjusted: α 0.05/3=0.017
HR, Hazard ratio; CI, Confidence interval

b

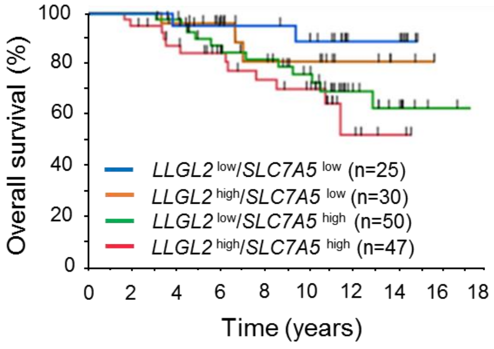

OS	*P*-value	HR	95%CI
LLGL2 ^{low}/*SLC7A5* ^{low} vs *LLGL2* ^{high}/*SLC7A5* ^{low}	0.52	1.7	0.28-13
LLGL2 ^{low}/*SLC7A5* ^{low} vs *LLGL2* ^{low}/*SLC7A5* ^{high}	0.10	3.2	0.88-21
LLGL2 ^{low}/*SLC7A5* ^{low} vs *LLGL2* ^{high}/*SLC7A5* ^{high}	0.03	4.5	1.2-29

*Bonferroni adjusted: α 0.05/3=0.017
HR, Hazard ratio; CI, Confidence interval
