# Supplementary material for: Co-expression effect of LLGL2 and SLC7A5 to predict prognosis in ERα-positive breast cancer
Source: Sci Rep. 2022 Oct 3;12:16515. doi: 10.1038/s41598-022-20225-4 (PMC9529905; doi:10.1038/s41598-022-20225-4)
Supplement: Supplementary file 2 — Supplementary Tables. [file 41598_2022_20225_MOESM2_ESM.pdf]

# Supplementary Tables

**Title: Co-expression effect of LLGL2 and SLC7A5 to predict prognosis in ERα-positive breast cancer**

**Author’s name:** Tomoka Hisada, Naoto Kondo, Yumi Wanifuchi-Endo, Satoshi Osaga, Takashi Fujita, Tomoko Asano, Yasuaki Uemoto, Sayaka Nishikawa, Yusuke Katagiri, Mitsuo Terada, Akiko Kato, Hiroshi Sugiura, Katsuhiro Okuda, Hiroyuki Kato, Masayuki Komura, Satoshi Morita, Satoru Takahashi, and Tatsuya Toyama

**Supplementary Table S1**

Association between *LLGL2* mRNA expression and clinicopathological characteristics in all breast cancer patients

| | All patients | *LLGL2* mRNA expression | | |
|---|---|---|---|---|
| | | Low | High | *P* value |
| | n (%) | n (%) | n (%) | |
| Patients | 624 | 337 | 287 | |
| Menopausal status | | | | 0.46 |
| Pre | 262 (42.0) | 146 (43) | 116(43) | |
| Post | 360 (57.7) | 189(56) | 171(57) | |
| Unknown | 2 (0.3) | 2 (1) | | |
| Tumor size | | | | 0.047 |
| ≤2cm | 240 (38.4) | 117 (34.7) | 123 (43) | |
| >2cm | 383 (61.4) | 219 (65.0) | 164 (57) | |
| Unknown | 1 (0.2) | 1 (0.3) | | |
| Nodal status | | | | 0.031 |
| Negative | 339 (54) | 196 (58) | 143 (50) | |
| Positive | 257 (41) | 125 (37) | 132 (46) | |
| Unknown | 28 (5) | 16 (5) | 12 (4) | |
| Grade | | | | 0.62 |
| 1 | 164 (26) | 89 (27) | 75 (26) | |
| 2 | 191 (31) | 98 (29) | 93 (32) | |
| 3 | 253 (41) | 141 (42) | 112 (39) | |
| Unknown | 16 (3) | 7 (2) | 9 (3) | |
| Histology | | | | 0.37 |
| IDC | 556 (89) | 301(89) | 255 (89) | |
| ILC | 27 (4) | 9 (3) | 18 (6) | |
| Others | 41 (7) | 27 (8) | 14 (5) | |
| ERα status | | | | 0.28 |
| Positive | 491 (78.7) | 259 (76.9) | 232 (81) | |
| Negative | 132 (21.1) | 77 (22.8) | 55 (19) | |
| Unknown | 1 (0.2) | 1 (0.3) | 0 (0) | |
| PgR status | | | | 0.93 |
| Positive | 442 (71) | 239 (71) | 203 (71) | |
| Negative | 181 (29) | 97 (29) | 84 (29) | |
| Unknown | 1 (0.2) | | | |
| HER2 status | | | | 0.34 |
| Positive | 82 (13) | 40 (12) | 42 (15) | |
| Negative | 518 (83) | 283 (84) | 235 (82) | |
| Unknown | 24 (4) | 14 (4) | 10 (3) | |
| Adjuvant therapy | | | | |
| ET alone | 265 (42) | 150 (45) | 115 (40) | |
| CT alone | 92 (15) | 62 (18) | 30 (10) | |
| ET+CT | 206 (33) | 92 (27) | 114 (40) | |
| None | 51 (8) | 26 (8) | 25 (9) | |
| Unknown | 10 (2) | 7 (2) | 3 (1) | |

ERα, estrogen receptor α; PgR, progesterone receptor; IDC, invasive ductal carcinoma; ILC, invasive lobular carcinoma; HER2, human epidermal growth factor receptor 2; ET, endocrine therapy; CT, chemotherapy.

**Supplementary Table S2**

Association between *LLGL2* mRNA expression and clinicopathological characteristics in ERα-breast cancer patients

| | All patients | *LLGL2* mRNA expression | | |
|---|---|---|---|---|
| | | Low | High | *P* value |
| | n (%) | n (%) | n (%) | |
| Patients | 491 | 259 | 232 | |
| Menopausal status | | | | 0.36 |
| Pre | 216 (44) | 119 (46) | 97 (42) | |
| Post | 275 (56) | 140 (54) | 135 (58) | |
| Tumor size | | | | 0.10 |
| ≤2cm | 207 (42) | 100 (38) | 107 (46) | |
| >2cm | 284 (58) | 159 (62) | 125 (54) | |
| Nodal status | | | | 0.007 |
| Negative | 258 (53) | 151 (59) | 107 (46) | |
| Positive | 209 (43) | 96 (37) | 113 (49) | |
| Unknown | 24 (4) | 12 (5) | 12 (5) | |
| Grade | | | | 0.67 |
| 1 | 160 (33) | 87 (34) | 73 (32) | |
| 2 | 153 (31) | 78 (30) | 75 (32) | |
| 3 | 166 (34) | 89 (34) | 77 (33) | |
| Unknown | 12 (2) | 5 (2) | 7 (3) | |
| Histology | | | | 0.12 |
| IDC | 438 (89.2) | 234 (90) | 205 (88) | |
| ILC | 26 (5.3) | 9 (3) | 17 (7) | |
| Others | 26 (5.3) | 16 (7) | 10 (4) | |
| Unknown | 1 (0.2) | 0 | 0 | |
| PgR status | | | | 0.09 |
| Positive | 426 (87) | 231 (89) | 195 (84) | |
| Negative | 65 (13) | 28 (11) | 37 (16) | |
| Unknown | 0 | 0 | 0 | |
| HER2 status | | | | 0.44 |
| Positive | 45 (9) | 21 (8) | 24 (10) | |
| Negative | 429 (87) | 227 (88) | 202 (87) | |
| Unknown | 17 (4) | 11 (4) | 6 (3) | |
| Adjuvant therapy | | | | |
| ET alone | 251 (51) | 141 (54) | 110 (47) | |
| CT alone | 29 (6) | 22 (8) | 7 (3) | |
| ET+CT | 173 (35) | 77 (30) | 96 (41) | |
| None | 32 (7) | 15 (6) | 17 (7) | |
| Unknown | 7 (1) | 5 (2) | 2 (1) | |
| Endocrine therapy | | | | |
| With tamoxifen | 272 (56) | 143 (55) | 129 (56) | |
| Without tamoxifen | 152 (31) | 75 (29) | 77 (33) | |
| None | 60 (12) | 36 (14) | 24 (10) | |
| Unknown | 7 (1) | 5 (2) | 2 (1) | |

PgR, progesterone receptor; IDC, invasive ductal carcinoma; ILC, invasive lobular carcinoma; HER2, human epidermal growth factor receptor 2; ET, endocrine therapy; CT, chemotherapy.

**Supplementary Table S3**

Univariate and multivariate analyses of factors associated with DFS and OS including *LLGL2* in all patients

| | | Univariate (DFS) | Univariate (OS) | Multivariate (DFS) | | Multivariate (OS) | |
|---|---|---|---|---|---|---|---|
| Variate | n (%) | *P*-value | *P*-value | *P*-value | HR (95% CI) | *P*-value | HR (95% CI) |
| Menopausal status | | | | | | | |
| Pre | 247 (44) | | | | | | |
| Post | 313 (56) | 0.49 | 0.44 | | | | |
| Tumor size | | | | | | | |
| ≤ 2 cm | 215 (38) | | | | | | 1 (Reference) |
| > 2 cm | 345 (62) | 0.041 | 0.003 | | | 0.064 | 1.53 (0.98, 2.39) |
| Nodal status | | | | | | | |
| Negative | 316 (56) | | | | 1 (Reference) | | 1 (Reference) |
| Positive | 244 (44) | < 0.0001 | < 0.0001 | < 0.0001 | 3.71 (2.64, 5.22) | < 0.0001 | 3.70 (2.45, 5.59) |
| Grade | | | | | | | |
| 1 and 2 | 323 (58) | | | | | | |
| 3 | 237 (42) | 0.039 | 0.022 | | | | |
| Histology | | | | | | | |
| IDC | 509 (91) | | | | | | |
| ILC | 19 (3) | 0.93 | 0.67 | | | | |
| Others | 32 (6) | 0.20 | 0.47 | | | | |
| ERα status | | | | | | | |
| Positive | 443 (79) | | | | 1 (Reference) | | 1 (Reference) |
| Negative | 117 (21) | 0.001 | 0.001 | < 0.0001 | 2.18 (1.53, 3.10) | < 0.0001 | 2.29 (1.52, 3.45) |
| HER2 status | | | | | | | |
| Negative | 484 (86) | | | | | | |
| Positive | 76 (14) | 0.042 | 0.042 | | | | |
| *LLGL2* mRNA expression | | | | | | | |
| Low | 299 (53) | | | | 1 (Reference) | | 1 (Reference) |
| High | 261 (47) | 0.026 | 0.10 | 0.083 | 1.33 (0.96, 1.82) | 0.17 | 1.30 (0.89, 1.89) |

ERα, estrogen receptor α; PgR, progesterone receptor; IDC, invasive ductal carcinoma; ILC, invasive lobular carcinoma; HER2, human epidermal growth factor receptor 2; ET, endocrine therapy; CT, chemotherapy.

**Supplementary Table S4**

Univariate and multivariate analyses of factors associated with DFS and OS including *LLGL2* in ERα-positive breast cancer patients

| Variate | n (%) | Univariate (DFS) *P*-value | Univariate (OS) *P*-value | Multivariate (DFS) *P*-value | Multivariate (DFS) HR (95% CI) | Multivariate (OS) *P*-value | Multivariate (OS) HR (95% CI) |
|---|---|---|---|---|---|---|---|
| Menopausal status | | | | | | | |
| Pre | 206 (46) | | | | | | |
| Post | 237 (54) | 0.29 | 0.20 | | | | |
| Tumor size | | | | | | | |
| ≤ 2 cm | 184 (42) | | | | | | 1 (Reference) |
| > 2 cm | 259 (58) | 0.14 | 0.011 | | | 0.043 | 1.53 (0.98, 2.39) |
| Nodal status | | | | | | | |
| Negative | 244 (55) | | | | 1 (Reference) | | 1 (Reference) |
| Positive | 199 (45) | < 0.0001 | < 0.0001 | < 0.0001 | 3.07 (2.05, 4.61) | < 0.0001 | 2.90 (1.76, 4.77) |
| Grade | | | | | | | |
| 1 and 2 | 287 (65) | | | | | | |
| 3 | 156 (35) | 0.39 | 0.11 | | | | |
| Histology | | | | | | | |
| IDC | 402 (91) | | | | | | |
| ILC | 18 (4) | 0.6 | 0.37 | | | | |
| Others | 23 (5) | 0.09 | 0.29 | | | | |
| HER2 status | | | | | | | |
| Negative | 402 (91) | | | | 1 (Reference) | | 1 (Reference) |
| Positive | 41 (9) | 0.19 | 0.038 | 0.04 | 1.84 (1.02, 3.29) | 0.004 | 2.58 (1.35, 4.95) |
| *LLGL2* mRNA expression | | | | | | | |
| Low | 233 (53) | | | | 1 (Reference) | | 1 (Reference) |
| High | 260 (47) | 0.002 | 0.007 | 0.012 | 1.80 (1.14, 2.84) | 0.011 | 2.15 (1.20, 3.86) |

IDC, invasive ductal carcinoma; ILC, invasive lobular carcinoma; HER2, human epidermal growth factor receptor 2; ET, endocrine therapy; CT, chemotherapy.

**Supplementary Table S5**

Association between *SLC7A5* mRNA expression and clinicopathological characteristics in ERα-positive breast cancer patients

| | All patients | *SLC7A5* mRNA expression | | |
|---|---|---|---|---|
| | | Low | High | *P* value |
| | n (%) | n (%) | n (%) | |
| Patients | 491 | 175 | 316 | |
| Menopausal status | | | | 0.058 |
| Pre | 216 (44) | 87 (50) | 129 (41) | |
| Post | 275 (56) | 88 (50) | 187 (59) | |
| Tumor size | | | | 1 |
| ≤2cm | 207 (42) | 74 (42) | 133 (42) | |
| >2cm | 284 (58) | 101 (58) | 183 (58) | |
| Nodal status | | | | 0.009 |
| Negative | 258 (53) | 106 (61) | 152 (48) | |
| Positive | 209 (43) | 61 (35) | 148 (47) | |
| Unknown | 24 (5) | 8 (4) | 16 (5) | |
| Grade | | | | 0.008 |
| 1 | 160 (33) | 65 (37) | 95 (30) | |
| 2 | 155 (32) | 63 (36) | 92 (29) | |
| 3 | 144 (29) | 43 (25) | 121 (38) | |
| Unknown | 12 (2) | 4 (2) | 8 (3) | |
| Histology | | | | 0.46 |
| IDC | 438 (89) | 152 (87) | 286 (91) | |
| ILC | 26 (5) | 10 (6) | 16 (5) | |
| Others | 27 (6) | 12 (7) | 14 (4) | |
| PgR status | | | | 1 |
| Positive | 426 (87) | 152 (87) | 274 (87) | |
| Negative | 65 (13) | 23 (13) | 42 (13) | |
| HER2 status | | | | 1 |
| Positive | 45 (9) | 17 (10) | 28 (10) | |
| Negative | 429 (87) | 153 (87) | 276 (87) | |
| Unknown | 17 (4) | 5 (3) | 12 (3) | |
| Adjuvant therapy | | | | |
| ET alone | 251 (51) | 103 (58) | 148 (47) | |
| CT alone | 29 (6) | 4 (2) | 25 (8) | |
| ET+CT | 173 (35) | 57 (33) | 116 (37) | |
| None | 31 (7) | 10 (6) | 21 (6) | |
| Unknown | 7 (1) | 1 (1) | 6 (2) | |
| Endocrine therapy | | | | |
| With tamoxifen | 272 (56) | 105 (60) | 167 (53) | |
| Without tamoxifen | 181 (31) | 59 (34) | 122 (37) | |
| None | 31 (12) | 10 (6) | 21 (7) | |
| Unknown | 7 (1) | 1 (0) | 6 (2) | |

PgR, progesterone receptor; IDC, invasive ductal carcinoma; ILC, invasive lobular carcinoma; HER2, human epidermal growth factor receptor 2; ET, endocrine therapy; CT, chemotherapy.

**Supplementary Table S6**

Association between *LLGL2* and *SLC7A5* mRNA co-expression and clinicopathological characteristics in all breast cancer patients

| | *LLGL2/SLC7A5* | | | | |
|---|---|---|---|---|---|
| | Low/Low<br>n (%) | High/Low<br>n (%) | Low/High<br>n (%) | High/High<br>n (%) | *P* value |
| Patients | 91 | 101 | 246 | 186 | |
| Menopausal status | | | | | |
| Pre | 49 (39) | 48 (48) | 97 (40) | 68 (37) | 0.026 |
| Post | 42 (61) | 53 (52) | 147 (60) | 118 (63) | |
| Tumor size | | | | | |
| ≤2cm | 31 (34) | 47 (46) | 86 (35) | 76 (41) | 0.162 |
| >2cm | 60 (66) | 54 (54) | 159 (65) | 110 (59) | |
| Unknown | | | 1 (0.4) | | |
| Nodal status | | | | | |
| Negative | 62 (68) | 56 (55) | 134 (54) | 87 (47) | 0.007 |
| Positive | 25 (28) | 41 (41) | 100 (41) | 91 (49) | |
| Unknown | 4 (4) | 4 (4) | 12 (5) | 8 (4) | |
| pStage TNM*1 | | | | | |
| I | 30 (33) | 32 (32) | 62 (25) | 47 (25) | 0.047 |
| II | 46 (51) | 48 (47) | 119 (48) | 91 (49) | |
| III | 11 (12) | 15 (15) | 51 (21) | 38 (21) | |
| Unknown | 4 (4) | 6 (6) | 14 (6) | 10 (5) | |
| Grade | | | | | |
| 1+2 | 66 (73) | 64 (63) | 121 (49) | 104 (56) | 0.0008 |
| 3 | 23 (25) | 35 (35) | 118 (48) | 77 (41) | |
| Unknown | 2 (2) | 2 (2) | 7 (3) | 5 (3) | |
| Histology | | | | | |
| IDC | 78 (87) | 89 (88) | 223 (91) | 166 (89) | 0.23 |
| ILC | 4 (4) | 6 (6) | 5 (2) | 12 (7) | |
| Others | 8 (9) | 6 (6) | 18 (7) | 8 (4) | |
| Unknown | | | 1 (0.4) | | |
| ERα status | | | | | |
| Positive | 84 (92) | 91 (90) | 175 (71) | 141 (76) | <0.001 |
| Negative | 7 (8) | 10 (10) | 70 (29) | 45 (24) | |
| Unknown | | | 1 (0.4) | | |
| PgR status | | | | | |
| Positive | 77 (85) | 79 (78) | 162 (66) | 124 (67) | 0.002 |
| Negative | 14 (15) | 22 (22) | 83 (34) | 62 (33) | |
| Unknown | | | 1 (0.4) | | |
| HER2 status | | | | | |
| Positive | 7 (8) | 16 (16) | 33 (13) | 26 (14) | 0.59 |
| Negative | 79 (87) | 83 (82) | 204 (83) | 152 (82) | |
| Unknown | 5 (5) | 2 (2) | 9 (4) | 8 (4) | |
| Adjuvant therapy | | | | | |
| ET alone | 58 (64) | 48 (47) | 92 (37) | 67 (36) | |
| CT alone | 6 (7) | 5 (5) | 56 (23) | 25 (13) | |
| ET+CT | 24 (26) | 38 (37) | 68 (28) | 76 (41) | |
| None | 3 (3) | 9 (9) | 23 (9) | 16 (9) | |
| Unknown | | 1 (1) | 7 (3) | 2 (1) | |

ERα, estrogen receptor α; PgR, progesterone receptor; IDC, invasive ductal carcinoma; ILC, invasive lobular carcinoma; HER2, human epidermal growth factor receptor 2; ET, endocrine therapy; CT, chemotherapy.

*1: AJCC stage

**Supplementary Table S7**

Univariate and multivariate analyses of factors associated with DFS and OS including *LLGL2*/*SLC7A5* in all patients

| | | Univariate (DFS) | Univariate (OS) | Multivariate (DFS) | | Multivariate (OS) | |
|---|---|---|---|---|---|---|---|
| Variate | n (%) | *P*-value | *P*-value | *P*-value | HR (95% CI) | *P*-value | HR (95% CI) |
| Age | | | | | | | |
| ≤ 50 | 236 (42) | | | | | | |
| > 50 | 324 (58) | 0.34 | 0.53 | | | | |
| Menopausal status | | | | | | | |
| Pre | 247 (44) | | | | | | |
| Post | 313 (56) | 0.49 | 0.44 | | | | |
| Tumor size | | | | | | | |
| ≤ 2 cm | 215 (38) | | | | | | 1 (Reference) |
| > 2 cm | 345 (62) | 0.041 | 0.003 | | | 0.06 | 1.53 (0.98, 2.39) |
| Nodal status | | | | | | | |
| Negative | 316 (56) | | | | 1 (Reference) | | 1 (Reference) |
| Positive | 244 (44) | < 0.0001 | < 0.0001 | < 0.0001 | 3.49 (2.48, 4.92) | < 0.0001 | 3.42 (2.26, 5.18) |
| Grade | | | | | | | |
| 1 and 2 | 323 (58) | | | | | | |
| 3 | 237 (42) | 0.039 | 0.022 | | | | |
| Histology | | | | | | | |
| IDC | 509 (91) | | | | | | |
| ILC | 19 (3) | 0.93 | 0.67 | | | | |
| Others | 32 (6) | 0.20 | 0.47 | | | | |
| ERα status | | | | | | | |
| Positive | 443 (79) | | | | 1 (Reference) | | 1 (Reference) |
| Negative | 117 (21) | 0.001 | 0.001 | 0.0003 | 1.94 (1.36, 2.78) | 0.001 | 1.99 (1.31, 3.01) |
| HER2 status | | | | | | | |
| Negative | 484 (86) | | | | | | |
| Positive | 76 (14) | 0.042 | 0.042 | | | | |
| *LLGL2/SLC7A5* | | | | | | | |
| Low/Low | 81 (14) | | | | 1 (Reference) | | 1 (Reference) |
| High/Low | 94 (17) | 0.004 | 0.032 | 0.013 | 2.95 (1.26, 6.92) | 0.051 | 3.12 (0.99, 9.75) |
| Low/High | 218 (39) | 0.0005 | 0.001 | 0.007 | 2.98 (1.35, 6.54) | 0.008 | 3.98 (1.42, 11.13) |
| High/High | 167 (30) | < 0.0001 | 0.0003 | 0.002 | 3.49 (1.58, 7.72) | 0.003 | 4.79 (1.70, 13.52) |

ERα, estrogen receptor α; PgR, progesterone receptor; IDC, invasive ductal carcinoma; ILC, invasive lobular carcinoma; HER2, human epidermal growth factor receptor 2.
